# Bio-detoxification of ricin in castor bean (*Ricinus communis* L.) seeds

**DOI:** 10.1038/s41598-017-15636-7

**Published:** 2017-11-13

**Authors:** Natália L. Sousa, Glaucia B. Cabral, Pabline M. Vieira, Aisy B. Baldoni, Francisco J. L. Aragão

**Affiliations:** 1 Embrapa Recursos Genéticos e Biotecnologia, PqEB W5 Norte, 70770-900 Brasília, DF Brazil; 2 Universidade de Brasília, Departamento de Biologia Celular, Campus Universitário, 70910-900 Brasília, DF Brazil; 3 Instituto Federal Goiano, Campus Urutaí, Laboratório de Biotecnologia, 75790-000 Urutaí, GO Brazil; 4 Present Address: Embrapa Agrossilvipastoril, Rod. dos Pioneiros MT-222, 78550-970 Sinop, MT Brazil

## Abstract

Ricin is a highly toxic ribosome-inactivating lectin occurring in the seeds of castor bean (*Ricinus communis* L.). Castor bean grows throughout tropical and sub-tropical regions and is a very important crop due to its high seed content of ricinoleic acid, an unusual fatty acid, which has several industrial applications. However, due to the presence of the toxin, castor bean can cause death after the exposure of animals to low doses of ricin through skin contact, injection, inhalation or oral routes. Aiming to generate a detoxified genotype, we explored the RNAi concept in order to silence the ricin coding genes in the endosperm of castor bean seeds. Results indicated that ricin genes were effectively silenced in genetically modified (GM) plants, and ricin proteins were not detected by ELISA. Hemagglutination activity was not observed with proteins isolated from GM seeds. In addition, we demonstrated that seed proteins from GM plants were not toxic to rat intestine epithelial cells or to Swiss Webster mice. After oil extraction, bio-detoxified castor bean cake, which is very rich in valuable proteins, can be used for animal feeding. Gene silencing would make castor bean cultivation safer for farmers, industrial workers and society.

## Introduction

Castor bean (*Ricinus communis* L.) is commercially cultivated due to the high quality and content (mainly ricinoleic acid) of its seed oil. Major producers are India, Mozambique, China and Brazil, responsible for 1.7 million, 68.9, 40.0 and 37.5 thousand tons, respectively (http://www.fao.org/faostat). India is the main oil exporter, and the United States, the European Union, and China import about 84% of the castor oil available on the international market^1,2^. Ricinoleic acid (12-hydroxy-*cis*-9-octadecenoic acid) confers higher stability and viscosity on castor bean oil when compared to other vegetable oils and makes it a highly valued material in the composition of lubricants, plastics, cosmetics, paints, varnishes, ethanol and biodiesel^2,3^. However, castor bean seeds contain ricin, which is a highly toxic storage 7 S lectin. Ricin is a dimeric glycoprotein constituted of A- and B-polypeptide chains covalently linked by a disulfide bond^4^. The A-chain is a ribosome-inactivating enzyme that specifically depurinates the first adenosine in the GAGA nucleotide sequence from the conserved loop on the 28 S rRNA subunit^5,6^. This modification impairs the formation of a critical rRNA stem-loop configuration, to which elongation factor 2 binds during the translocation step of translation. The B-chain binds specifically to cell surface glycoproteins or glycolipids and facilitates the movement of the A-chain into animal cells. One A-chain molecule of ricin is able to irreversibly inactivate one thousand ribosomes per minute, impairing protein synthesis and causing cell death^7^. Castor bean seeds also contain the ricin homologue *R. communis* agglutinin (RCA_120_), which is a tetrameric protein composed of two A-chains (90% similar to the ricin A-chain) and two B-chains (84% similar to the ricin B-chain). RCA_120_ presents a reduced toxicity and a strong hemagglutinin of mammalian red blood cells^8^. Antibodies produced against ricin generally cross-react with RCA_120_^9^.

Seeds accumulate significant amounts of ricin 40 days after pollination^10^, ranging from 1.6 mg to 32 mg of ricin per gram of mature seed^11–13^. Ricin is synthesized as a precursor polypeptide (preproricin) of 64.1 kDa, encoding both A- and B-chains, which is converted into a 61.6 kDa proricin by cleavage of the N-terminal signal peptide, during transference to the lumen of the endoplasmic reticulum. In the vacuoles, propeptides are cleaved, and the ricin accumulates in protein bodies as a 58.8 kDa mature protein^14^.

Acute toxicity studies of ricin carried out with mice determined the lethal dose (LD_50_) values as 2.4 to 36 µg/kg and 21 to 30 mg/kg through intraperitoneal and oral routes, respectively^15^. The LD_50_ for humans is estimated to be between 1 and 20 mg/kg of body weight, and is much more toxic if the exposure is by inhalation (1–10 µg/kg of body weight)^15^. Symptoms include diarrhea, anorexia, abdominal pain, weakness, low appetite, cramps, and soft dark feces. Ricin could also be harmful to workers exposed to castor bean grains by both inhalation and skin contact during industrial processing. In addition, the US Center for Disease Control and Prevention characterized ricin as a category B priority biothreat agent that might be used for bioterrorism purposes^16–18^.

Castor cake, which is the remaining industrial sub-product generated after extraction of the oil from castor bean grains, is rich in valuable proteins and fiber and could be used as animal feed. Due to the presence of ricin, it is generally used only as an organic fertilizer. However, a number of domestic animals have presented intoxication and death after ingesting fertilizer containing castor cake^15^. Development of several castor cake detoxification methods has been achieved with limited success. It has been demonstrated that fermentation procedures with microorganisms, such as *Paecilomyces variotii* and *Aspergillus niger*, were able to detoxify castor bean residue separated during the process of biodiesel production^19,20^. In addition, castor cake detoxification has been achieved by chemical treatment with calcium compounds (calcium oxide and calcium hydroxide)^20^. From the economic point of view, these processes are still not practical and efficient enough to be used on a large scale^3,20^.

Castor bean genome sequencing revealed several putative genes in the ricin family, including potential pseudogenes or gene fragments, forming clusters in the plant genome^21^. Despite the difficulties in determining whether some shorter genes or pseudogenes are functional, at least seven full-length genes encode proteins with the ribosome-inactivating and lectin domains^21,22^. This might explain the difficulty in generating detoxified genotypes using classical mutation techniques. Consequently, current castor bean varieties with a lower content of ricin (70–75% less) are still very toxic to mammals^3,23^. Based on these facts, we explored the RNA interference (RNAi) concept to silence the ricin gene in castor bean seeds in order to generate a non-toxic castor bean genotype. RNAi is a post-transcriptional gene silencing mechanism that regulates the expression of protein-coding genes. Constructs to express self-complementary RNA transcripts form a dsRNA, which is processed into small interfering RNAs (siRNAs). These siRNAs trigger a sequence-specific mRNA degradation, leading to gene silencing^24^.

## Results and Discussion

**Figure 1:**
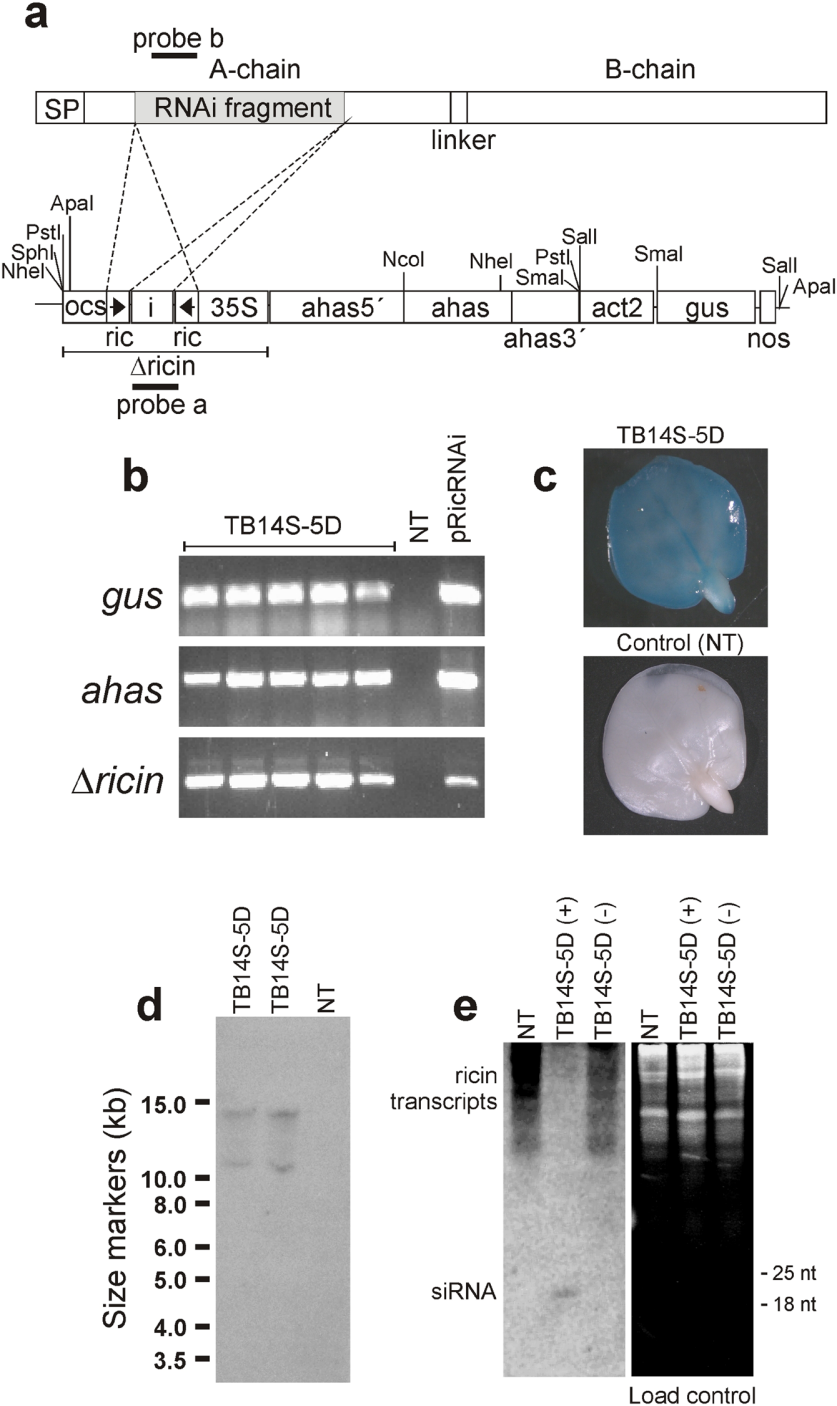
Engineering bio-detoxified castor bean (*R. communis* L.) seeds by silencing of the ricin genes. (**a**) A 460 bp fragment from the ricin A-chain gene was cloned in sense and antisense orientations under the control of the 35 S CaMV promoter for the construction of the intron-hairpin RNAi cassette (Δ*ricin*). The pRicRNAi vector also contained the reporter *gus* gene and the mutated *Arabidopsis thaliana* ahas gene, which confers tolerance to imidazolinones. (**b**) PCR analyses confirmed the presence of the *gus*, *ahas* and Δ*ricin* transgenes. (**c**) Expression of the *gus* gene in transgenic embryos (TB14S-5D). Non-transgenic (NT) embryos did not show GUS expression. (**d**) Southern blot analysis revealed the presence of two copies of the Δ*ricin* integrated into the genome of two transgenic plants (TB14S-5D). No signal was observed in non-transgenic plants (NT). Genomic DNA was hybridized with probe a. Molecular size markers are indicated on the left. (**e**) Northern blot analysis was carried out with the fragment of ricin probe (solid bar) and shows the presence of the ricin siRNA and absence of ricin gene transcript in transgenic seed [TB14S-5D (+)]. In contrast, ricin siRNA transcripts were absent and ricin RNA transcripts were present in non-transgenic (NT) or negative segregating seeds [TB14S-5D (−)]. Full-length Southern and Northern blots and gel images (**b**,**d**,**e**) are presented in Supplementary Figure 2.

An intron-hairpin vector was designed, in which a 460 bp fragment of the A-chain ricin gene was directionally cloned in sense and antisense (Δricin cassette) to generate dsRNA during transcription (Fig. 1a). In addition, the vector contained the *Arabidopsis thaliana ahas* gene (which confers tolerance to the herbicide imazapyr) and the *gus* gene (used for screening transformants). Of 270 embryonic axes bombarded with the vector pRicRNAi (Fig. 1a), four primary transgenic lines (R_0_) were produced, which represents a transformation efficiency of 0.85%.

Transgenic plants were analyzed for the *gus* gene expression and results have shown that all lines were transgenic. However, only one line (named TB14S-5D) transferred the transgenes to the progeny. The presence of the *gus*, *ahas* and Δ*ricin* transgenes was confirmed by PCR (Fig. 1b). The chi-square test revealed a Mendelian segregation ratio of 3:1 in the T_1_ generation (26 positive: 12 negative; Χ^2^ = 0.87; P = 0.35; df = 1). The line TB14S-5D presented a strong GUS histochemical activity and became visibly blue in 20–30 minutes in leaves, endosperms and embryonic axes (Fig. 1c). It will be useful to determine, even under field conditions, whether a specific variety is genetically modified. This is important for the safer use of the event TB14S-5D.

Southern blot analysis of transgenic castor bean plants revealed the presence of the Δ*ricin* interfering cassette (Fig. 1d). Since the pRicRNAi vector has a unique NcoI restriction site (Fig. 1a), Southern analysis allowed us to estimate the presence of two integrated copies of the Δ*ricin* cassette in the genome of the transgenic plants. DNA isolated from non-transformed plants did not hybridize with the probe (Fig. 1d).

It has been shown that intron-spliced constructs under control of the 35 S CaMV promoter can induce silencing of endogenous genes in seed tissues with 90 to 100% efficiency^25,26^. Our Northern blot analyses revealed that ricin transcripts were detected only in the endosperm of non-transgenic and negative-segregating seeds (Fig. 1e). mRNAs of several sizes were observed in non-transgenic seeds, probably due to the fact that 19 members of ricin/RCA_120_ genes and pseudogenes containing the A-chain sequence were found in the castor bean genome. These fragments could vary from 0.8 to 1.7 kb^21^. In contrast, it was difficult to detect transcripts corresponding to ricin genes in the endosperm of transgenic seeds. Accordingly, siRNA molecules corresponding to ricin sequences were only observed in transgenic seeds (Fig. 1e). These results indicate that ricin members as well as RCA coding genes were effectively silenced.

**Figure 2:**
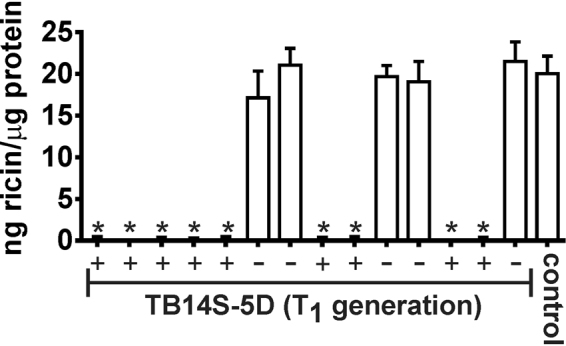
Detection of ricin in bio-detoxified event TB14S-5D. ELISA was used to detect and quantify ricin in the endosperm of castor bean seeds. Ricin was detected in non-transgenic seeds (control, wild type plants) and in the negative segregating seeds of the T_1_ generation [marked with (−)].However, ricin could not be detected in positive transgenic seeds [marked with (+)]. Asterisks represent significant differences compared to control (*P* < 0.01, *n* = 9).

ELISA was used to detect and quantify ricin in segregating seeds of the transgenic line TB14S-5D. The results showed that seeds from the cv. EBDA-MPA-34 (wild type) presented 20 ng ricin/µg of total protein. Segregating seed, which does not contain the transgenes, presented statistically similar quantities of ricin when compared to the control (non-transgenic plants). In contrast, ricin was not detectable in transgenic seeds (Fig. 2). Considering that the antibody raised against the ricin A-chain cross-reacts with its homologue RCA_120_, our results suggested that both ricin and RCA_120_ were silenced. This is expected because the ricin fragment used for the Δ*ricin* interfering cassette construction has 94% identity with the RCA_120_ coding gene.

**Figure 3:**
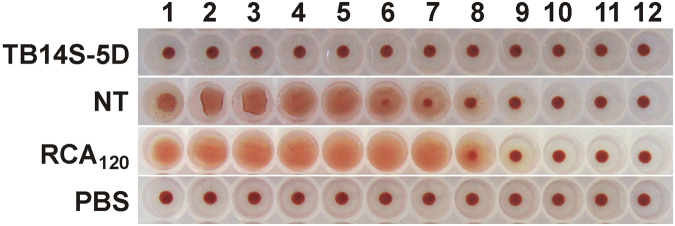
Proteins from transgenic event TB14S-5D do not agglutinate red blood cells. Proteins from transgenic (TB14S-5D) and non-transgenic (NT) seeds were tested for their capacity to hemagglutinate red blood cells (RBC, 2% suspension). Protein concentration was serially diluted by a ratio of 0.5 from wells 1 to 12, starting with 2.5 µg/µL. RCA_120_ (starting with 0.1 µg/µL) was used as a positive control and PBS was a negative control. Agglutinated RBC formed a diffuse mat, whereas non-agglutinated RBC sediment formed a dot at the bottom of the well.

The hemagglutination assay was performed with total proteins isolated from the endosperm of transgenic and non-transgenic castor bean seeds. A strong hemagglutination was visible with proteins isolated from non-transgenic plants at the concentration of 2.5 µg/µL of total protein and was evident until the concentration of 19 ng/µL of total protein (Fig. 3). In contrast, no visible hemagglutination activity was observed with proteins isolated from transgenic seeds, even at a higher protein concentration (approximately 131 times more concentrated). In addition, agglutination activity was not observed in cow blood cells incubated with PBS (blank). Purified RCA_120_ presented strong hemagglutination activity until the lowest concentration of 0.39 ng/µL (Fig. 3). Ricin has been described as a weak hemagglutinin, whereas RCA_120_ presents a strong hemagglutination activity^27^. However, a characteristic hemagglutination activity (with a titer of 16) has been observed for one ricin isoform (ricin III)^28^. Nevertheless, the fact that no hemagglutination activity was observed with proteins from the transgenic line TB14S-5D confirms that both RCA_120_ and ricin were efficiently silenced.

**Figure 4:**
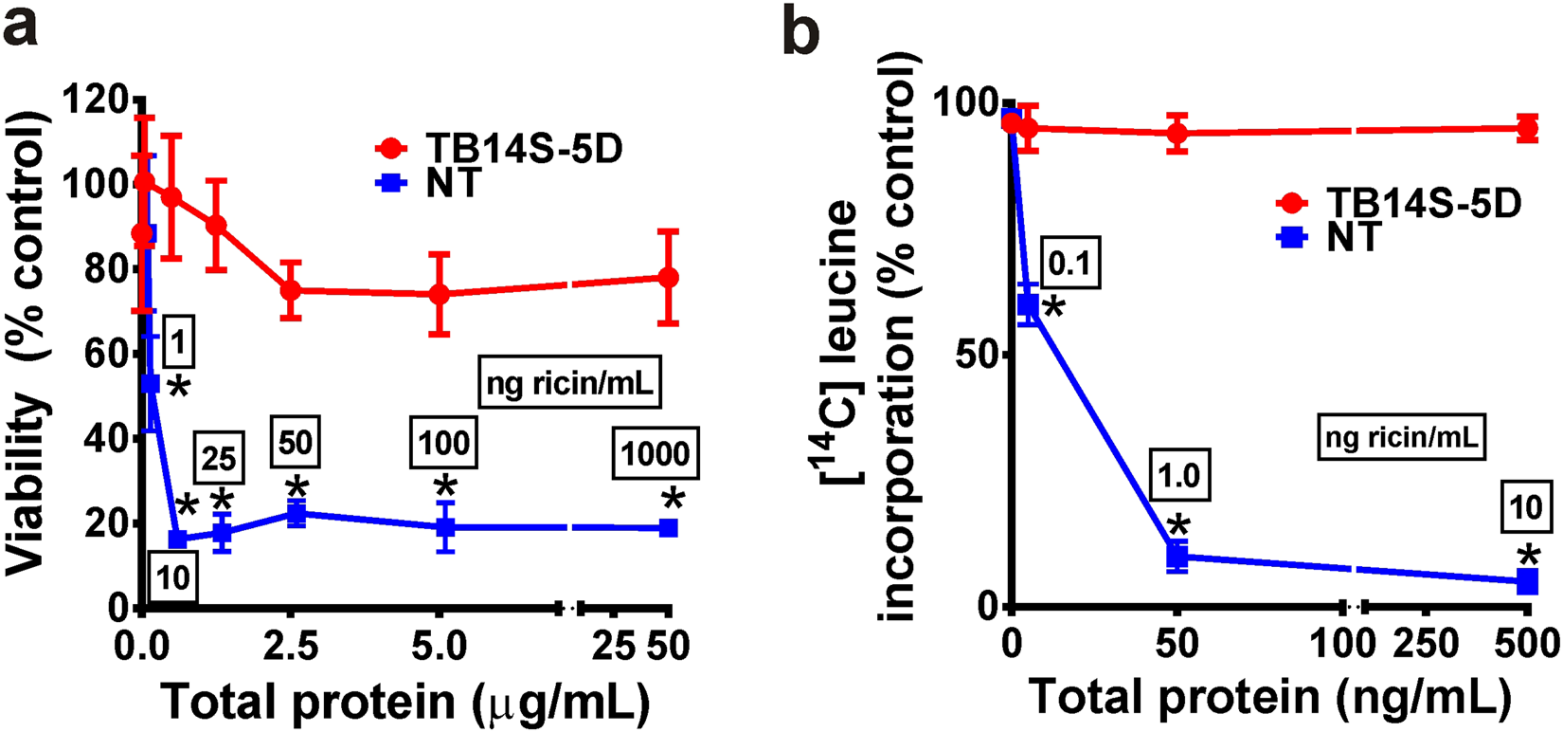
Toxicity performance of transgenic event TB14S-5D. (**a**) Rat small intestine epithelial cells (IEC-6) were incubated with proteins isolated from transgenic (TB14S-5D) and non-transgenic seeds (NT). In proteins from the non-transgenic seeds, 0 to 50.0 µg total protein/mL contained 0 to 1000 ng ricin/mL (numbers in boxes). There was no statistical difference between the values observed in the TB14S-5D viability curve. Values are expressed as number of viable cells as a percentage of control cells (cultivated only in the DMEM medium). *n* = 9. Asterisks represent significant statistical differences compared to control (*P* < 0.01). (**b**) Inhibition of protein synthesis was quantified in IEC-6 cells incubated with total proteins isolated from non-transgenic (NT) and transgenic TB14S-5D seeds. In proteins from the non-transgenic seeds, 0 to 500 ng total protein/mL, contained 0 to 10 ng ricin/mL (numbers in boxes). Data were expressed as the percentage of incorporated L-[^14^C(U)]leucine into proteins of the IEC-6 cells relative to the control (cells incubated with DMEM medium). *n* = 9. Asterisks represent significant statistical differences compared to control (*P* < 0.01).

IEC-6 (rat intestine epithelial cells) were incubated for 24 h with total proteins isolated from seed endosperm of transgenic and non-transgenic plants. The viability of cells exposed to proteins isolated from non-transgenic plants, containing 1 and 10 ng ricin/mL, was reduced to 53% and 16%, respectively (Fig. 4a). However, cells exposed to the equivalent amount of proteins from the transgenic seeds maintained their viability at 97% (at 0.5 µg protein/mL) and 78% at the higher concentration of protein, at 50 µg protein/mL (Fig. 4a and Supplementary Fig. 1). There were no statistical differences between the viability values of 97% and 78%. These results were corroborated by the fact that protein synthesis was 40% and 90% inhibited by cells cultivated for 5 h with proteins from non-transgenic seeds containing 0.1 and 1 ng ricin/mL, respectively (Fig. 4b). However, no inhibition was observed in cells cultivated in the presence of the equivalent amount of proteins isolated from transgenic seeds, even at the highest total protein concentration (Fig. 4b).

**Figure 5:**
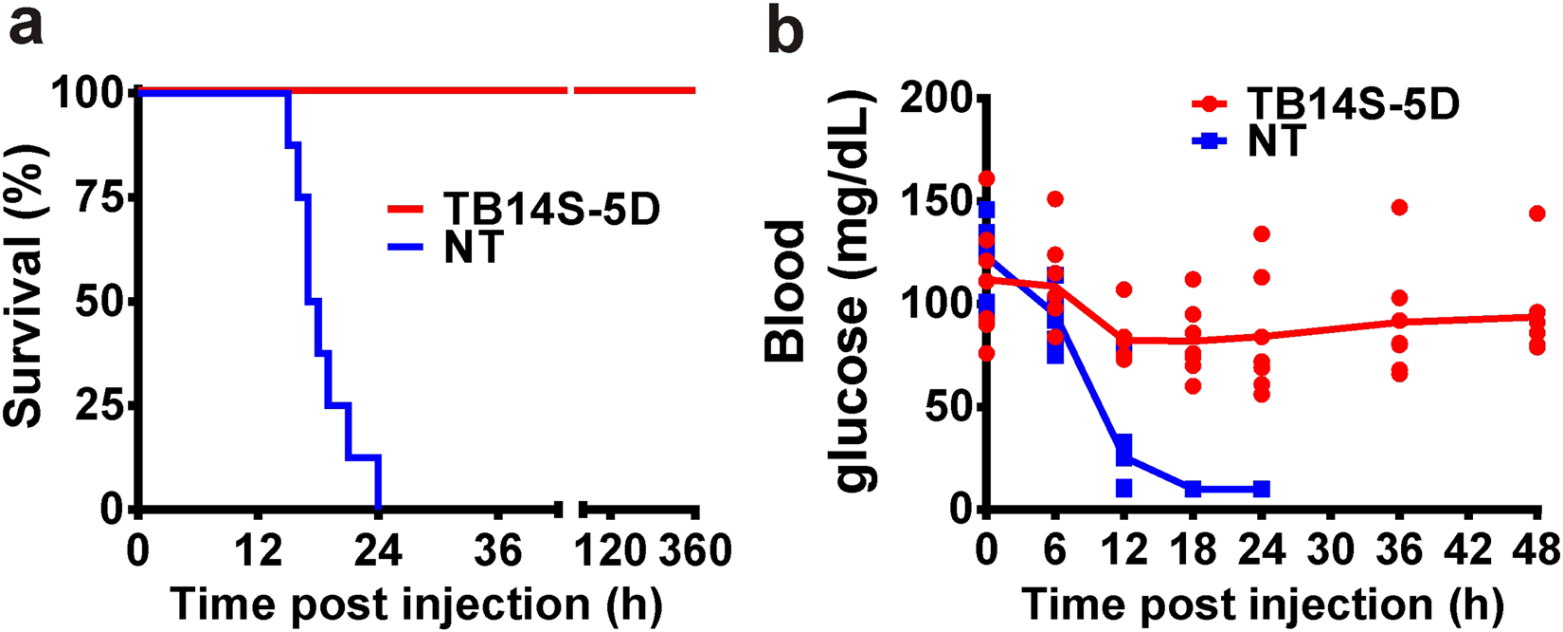
Ricin toxicosis (lethal challenge assay) evaluation in Swiss Webster mice. (**a**) Swiss albino mice were injected intraperitoneally with 100 µL of a solution of total protein extracted from non-transgenic (NT) seeds (20 µg protein/g body weight; 552 µg ricin/kg body weight) and the equivalent amount of protein (20 µg protein/g body weight) isolated from transgenic TB14S-5D seeds. Comparison of survival curves with log rank test yielded statistical significance of *P = *0.0145, *n* = 7. (**b**) Effect of intraperitoneal administration of proteins from transgenic (TB14S-5D) and non-transgenic (NT) seeds on blood glucose concentration was evaluated for a period of 48 h. *n* = 7.

Swiss Webster mice were treated with an intraperitoneal administration of ricin in order to measure ricin toxicosis (lethal challenge assay)^29^. We performed ricin challenge by injecting mice with total proteins isolated from the event TB14S-5D and non-transgenic seeds. As expected, all animals that were submitted to intraperitoneal injection of 20 µg protein/g body weight from wild type seeds (552 µg ricin/kg body weight) died within the first period of 24 h (Fig. 5a). However, animals injected with the equivalent amounts of total proteins isolated from seeds of event TB14S-5D survived with no visible ricin toxicosis symptoms (diarrhea, weakness, low appetite, soft dark feces and weight loss). It has been suggested that the mechanism of death in mice, after systemic administration of ricin, could be marked by hypoglycemia^29^. Indeed, a remarkable decrease was observed in blood glucose concentration in animals injected with proteins from non-transgenic seeds. However, there was no significant alteration in blood glucose of animals for a period of 60 h after injection with proteins from transgenic seeds (Fig. 5b). Animals were monitored for an additional period of seven days and no death was registered for those injected with proteins from event TB14S-5D.

Considering that the LD_50_ values in the literature range from 2.4 to 36 µg/kg, the ricin dose administrated in the *in vivo* toxicity assay was from 15 to 230 times the LD_50_ values for Swiss mice. Taking into consideration that the lethal dose for oral ingestion is approximately 1000–fold higher than by intraperitoneal injection, it is possible to predict that these animals would be able to consume up to 52% their body weight with TB14S-5D-derived castor bean cake, without acute intoxication. Usually, cows, sheep and goats consume only 1 to 2% of their body weight per day with protein sources, such as soybean ingredients. Thus, castor bean cake would be an excellent source of protein in animal feedstuffs^3,15^. Without toxic ricin and RCA_120_, castor meal might be widely used as an alternative to corn and soybean meal, which are typically the main ingredients of cattle, poultry and swine diets. Indeed, long-term studies have evaluated the use of physically or chemically detoxified castor bean cake for feeding cows, chickens and sheep, and no adverse effects have been observed^3,30–33^. Further studies should be carried out in order to determine the performance of the bio-detoxified castor bean cake in animal feeding.

A number of pieces of evidence support the idea that ricin plays a role in plant defense against pathogens and insects^34^. Moreover, the differential expression patterns observed for the ricin type I and type II members during seed development raised the hypothesis that ricin could have biological functions other than defense against predators^35^. The bio-detoxified event TB14S-5D could be used to study the ecological role of ricin and RCA_120_. Further studies should be carried out in order to determine the impact of ricin bio-detoxification of seeds on the cultivation of event TB14S-5D under field conditions.

Collectively, our results demonstrated that silencing ricin genes in castor bean seeds was effective and generated a bio-detoxified genotype. Castor bean can be cultivated under unfavorable environments (dry areas and impoverished soils), where farmers do not have many options, especially in a scenario of rising temperatures and reduced water sources. After oil extraction, the remaining processed material could be used as animal feed, due to its high nutritional value^3,33,36^. The bio-detoxified line TB14S-5D will make castor bean cultivation, harvest and post-harvest processing much safer. In addition, it will reduce the probability that commercial cultivation might be used to produce bioweapons. This biotechnology will have a major impact on the cultivation of castor bean, a plant already adapted to suboptimal environments, with a consequent positive effect on manufacturing ricinoleic acid-based goods and livestock production.

## Methods

### RNAi construct

A fragment of 460 bp from *R. communis* ricin A chain gene (position 129 to 589; GenBank accession number DQ661048) was amplified by PCR using the primer pair RcRinR [5′-GAAGCTTGGTACCTAATTCTCGTGCGCAT-3; including the sites for HindIII and KpnI (underlined)] and RcRinf [5′-GTCTAGACTCGAGACATGAAATACCAGTGTTGC-3; including the sites for XbaI and XhoI (underlined)]. PCR was carried out according to Bonfim *et al*.^37^. The amplified fragment was cloned into the pGEMT-Easy vector and sequenced. The ricin A-chain gene fragment was excised from pGEMT-Easy with XhoI/KpnI and HindIII/XbaI and inserted into the pKannibal vector^26^ interfering cassette (de novo synthesized by Epoch Life Science Inc., USA) in sense and antisense orientations, generating the ricin-interfering cassette (Δ*ricin*) (Fig. 1). The Δ*ricin* was transferred to the vector pAG1^38^, generating the pRi cRNAi which was used to transform castor bean. pAG1 contained the *gus* gene under the control of the *act2* promoter and the mutated *ahas* gene that confers tolerance to imazapyr^38^.

### Castor bean transformation

For transformation, mature seeds (elite cv. EBDA-MPA-34) were surface sterilized in 2.5% sodium hypochlorite and 0.05% Tween 20 for 30 min, and then rinsed three times in sterile distilled water. Then, seeds were soaked in distilled water for 16 h. The embryonic axes were excised from the seed surface and sterilized with 0.5% sodium hypochlorite for 10 minutes. Embryos were transferred to shoot induction medium [SIM; MS medium containing 300 mg/L casein, 100 mg/L thiamine, 0.5 mg/L thidiazuron (TDZ), 0.05 mg/L indoleacetic acid (IAA), 3% sucrose and 1.4% agar (Sigma), pH 4.0]. After 48 h, the apical meristems were exposed by removing the primary leaves. Embryonic axes were cultivated for an additional 16 h in SIM, and apical meristems were bombarded with tungsten particle carrying the vector pRicRNAi according to Rech *et al*.^39^. After bombardment, embryonic axes were maintained in SIM for one day in the dark and 28 °C. Then, explants were transferred to SIM supplement with 100 mg/L *myo*-inositol and 150 nM imazapyr. After one week the explants were transferred to MS medium supplemented with 1 mg/L zeatin, 0.1 mg/L IAA, 300 mg/L casein, 100 mg/L thiamine, 100 mg/L *myo*-inositol, 150 nM imazapyr, 3% sucrose and 1.4% agar, pH 4.0. After 10 days, embryos were transferred to elongation medium (MS medium containing 1 mg/L IAA, 1 mg/L gibberellic acid (GA_3_), 300 mg/L casein, 100 mg/L thiamine, 100 mg/L *myo*-inositol, 200 nM imazapyr, 3% sucrose and 1.4% agar, pH 4.0). As soon as the elongating shoots reached 2-3 cm in length, they were transferred to the rooting medium (MS medium supplemented with 1 mg/L indolebutyric acid (IBA), 5 µM AgNO_3_, 300 mg/L casein, 100 mg/L thiamine, 100 mg/L *myo*-inositol and 50 nM imazapyr, 3% sucrose and 1.4% agar, pH 4.0). Plantlets that reached 3-4 cm in length were acclimatized according to Rech *et al*.^39^. Except when specified, explants were cultured at 26 °C with 16-h photoperiod (140 µmols/m^2^/s).

### Screening of transgenic plants by the GUS histochemical assay

The GUS histochemical assay was carried out using tissues from regenerating shoots, acclimated plants and their progenies (leaves, endosperms and embryonic axes), according to Jefferson *et al*.^40^.

### Progeny analysis

The analysis of the T_1_ generation transformants was conducted by amplifying the introduced foreign genes (Δ*ricin*, *gus* and *ahas*) by PCR and by GUS histochemical assay analysis of leaves of plants pollinated by non-transgenic plants. PCR was carried out according to Bonfim *et al*.^37^ using the primer pair AHASP124 (ACTAGAGATTCCAGCGTCAC)/AHAS500C (GTGGCTATACAGATACCTGG) to amplify 685 bp within the *ahas* gene, the primer pair GUS671C (ATCACGCAGTTCAACGCTGAC)/GUS251 (TTGGGCAGGCCAGCGTATCGT) to amplify 421 bp within the *gus* gene, and the primer pair PSIUINTF (GAACCCAATTTCCCAACTG)/PSIUINTR (AGGTACCCCAATTGGTAAGGA) to amplify 798 bp within the Δ*ricin* cassette. Chi-square (χ2) analyses were performed to determine whether the observed segregation ratio was consistent with a Mendelian ratio in the T_1_ generation.

### Southern blot analysis

Genomic DNA isolation and hybridization were carried out as previously described^41^. The hybridization was carried out using a PCR-generated probe (probe a; Fig. 1a), labeled with [α^32^P] dCTP (110 TBq/mol) using the DecaLabel DNA labeling kit (Thermo Scientific) according to the manufacturer’s instructions. PCR was carried out according to Bonfim *et al*.^37^ with the primer pair PSIUINTF/PSIUINTR to amplify a 798 bp fragment corresponding to the *pdk* intron from the ricin-interfering cassette (Δ*ricin*).

### RNA analysis

Total RNA (30 μg) was isolated from endosperm of immature (50 days after pollination) seeds. RNA analyses were carried out according to Aragão *et al*.^42^. RNA was hybridized with a DNA probe corresponding to the 148 bp fragment PCR amplified using the primer pair Ric149RNAiF (GTAGCCGACCACATATGCATTG)/RcRinf: (GAGACATGAAATACCAGTGTTGC), within the A-chain ricin gene. Probes were labeled with ^α^32 P dCTP using the DecaLabel DNA Labeling Kit (Thermo Scientific) according to the manufacturer’s instructions. Hybridization and post hybridization washes were conducted as described^43^.

### Quantification of ricin content

Quantification of ricin content in mature seeds was carried out using ELISA according to Baldoni *et al*.^10^. For protein extraction, 200 mg of tissue (endosperm) was ground in liquid nitrogen and vortexed in 600 µL of sample buffer (PBS) for 30 min at 4 °C. The mixture was centrifuged at 20,800 g for 60 min at 4 °C and the aqueous phase collected. Total protein was quantified using the Quick Start Bradford Protein Assay (Bio-Rad Laboratories). For ricin detection, goat antiserum (Santa Cruz Biotechnology) was used, raised against a peptide located at the N-terminus of the ricin precursor. A standard curve was produced using purified ricin A (Sigma, L9514). The limit of detection was determined as 80 pg/µg total protein in the 50 µL-well. Absorbance was measured in a microplate reader (Bio-Rad) at 405 nm.

### Hemagglutination assay

Hemagglutination assay was carried out in 96-well microtiter plates. Total proteins from endosperm of transgenic and non-transgenic seeds were isolated as previously described. Each well contained 50 µL phosphate-buffered saline (PBS) and 50 µL of RCA_120_ were added (initial concentration of 0.1 µg/µl), 50 µL total proteins isolated from transgenic and non-transgenic castor bean endosperm serially diluted (initial concentration of 2.5 µg/µL) and 50 µL PBS (blank). Fifty microliters of a 2% suspension (diluted in 0.15 M NaCl) of cow (*Bos indicus*) red blood cells were added to each well and gently mixed. Plates were incubated at room temperature for 2 h and results were recorded. The titer was expressed as the reciprocal of dilution factor of the last well showing hemagglutination activity. Samples were observed using an inverted microscope.

### Cytotoxicity assay

IEC-6 (rat small intestine epithelial cells) were maintained at 37 °C under 5% CO_2_ atmosphere in Dulbecco’s Modified Eagle’s Minimum Essential Medium (DMEM, Invitrogen) supplemented with 10% (v/v) fetal calf serum, 2mM L-glutamine, 4.5 g/L glucose, 5 mg/L insulin, 100 UI/L penicillin and 50 µg/L streptomycin. The cells were grown in 75 cm^2^ vented tissue culture flasks. Culture medium was changed every three days. Cells were seeded on 24-well tissue culture plates, grown to confluence. Total protein isolated from endosperm of transgenic and non-transgenic seeds was added to the well to make the final concentration of ricin of 0, 1, 10, 25, 50 100 and 1,000 ng ricin/mL. Equivalent amounts of total proteins were used for the transgenic event TB14S-5D (0, 0.05, 0.5, 1.25, 2.5, 5.0 and 50.0 µg total protein/mL). Cells were incubated for 24 h, and the number of viable cells was determined according to Cheah *et al*.^44^. Data were expressed as number of viable cells as a percentage of control cells.

### Protein synthesis inhibition assay

IEC-6 cells (2 × 10^4^ cells/mL) in 100 µL Eagle’s MEM medium were added to the wells of 96-well microtiter plates and incubated at 37 °C under 5% CO_2_ atmosphere. To each well was added total protein isolated from endosperm of non-transgenic (NT) seeds containing 0, 0.1, 1 and 10ng/mL ricin. Equivalent amounts of protein were used for transgenic event TB14S-5D (5, 50 and 500 ng total protein/mL). Plates were incubated for 5 h and pulsed for 2 h after addition of 50 µL of medium containing L-[^14^C(U)]leucine (0.1 µCi/ml, specific activity >300 mCi/mMole). After washing the cells in PBS followed by 5% TCA they were lysed in 0.5 M NaOH and the precipitate was collected. Paper filters were counted using a scintillation counter. Data were expressed as the percentage of incorporated L-[^14^C(U)]leucine relative to the control (cells cultivated in DMEM medium).

### Acute toxicity assay

Healthy young adult outbred Swiss Webster mice (8 weeks old and weighing 30-45 g) obtained from the Laboratório de Pesquisas Biológicas (Instituto Federal Goiano, Brazil) were randomly allocated to treatment groups. All animals were housed in plastic cages (40 cm × 30 cm × 16 cm), in air-conditioned rooms at 22 ± 2 °C and 50 ± 10% of relative humidity, with a 12-hour light-dark natural cycle. Food (appropriate commercial rodent diet Nuvilab CR-1) and water were given *ad libitum*. Groups of seven animals were treated by intraperitoneal injection of 100 µL of total protein solution extracted from transgenic and non-transgenic castor bean seeds as previously described (20 µg protein/g body weight; 552 µg ricin/kg body weight). Doses were estimated based on previous studies performed to determine the maximum tolerated dose. For transgenic castor bean seeds the concentration of 20 µg protein/g body weight was considered. Mice were monitored for an additional period of seven days for signs of intoxication or death. Procedures were carried out following the international ethical principles for use of animals in testing and authorized by the Animal Experimentation Ethics Committee (CEUA-Instituto Federal Goiano; approval No. 2812220617). Blood glucose concentration was measured using a glucometer (Accu-Chek Active, Roche Diagnostics) according to the manufacturer’s instructions.

### Statistics

Data were expressed as the mean ± SEM of at least three independent experiments. Differences were considered statistically significant with *P* values of <0.01 using two-way ANOVA and Tukey’s multiple range test. Survival analyses of the Acute toxicity assay were performed using the log-rank (Mantel-Cox) test and Gehan-Breslow-Wilcoxon test (*P* value = 0.014). The GraphPad Prism Software (version 6.02) was used.

## Electronic supplementary material

Supplementary Information
