# Supplementary material for: Bio-detoxification of ricin in castor bean (Ricinus communis L.) seeds
Source: Sci Rep. 2017 Nov 13;7:15385. doi: 10.1038/s41598-017-15636-7 (PMC5684206; doi:10.1038/s41598-017-15636-7)
Supplement: Supplementary file 1 — Supplementary Information [file 41598_2017_15636_MOESM1_ESM.pdf]

## Suplementary Information

# Bio-detoxification of ricin in castor bean (*Ricinus communis* L.) seeds

Natália L. Sousa [1,2], Glaucia B. Cabral [1], Pabline M. Vieira [3], Aisy B. Baldoni[1,4], Francisco J. L. Aragão[1]*

[1] Embrapa Recursos Genéticos e Biotecnologia, PqEB W5 Norte, 70770-900, Brasília, DF, Brazil.

[2] Universidade de Brasília, Departamento de Biologia Celular, Campus Universitário, 70910-900, Brasília, DF, Brazil.

[3] Instituto Federal Goiano, Campus Urutaí, Laboratório de Biotecnologia, 75790-000, Urutaí, GO, Brazil.

[4] Current address: Embrapa Agrossilvipastoril, Rod. dos Pioneiros MT-222, 78550-970, Sinop, MT, Brazil.

*To whom correspondence may be addressed. Tel: +55 61 34484777; E-mail addresses: francisco.aragao@embrapa.br, fjlaragao@gmail.com

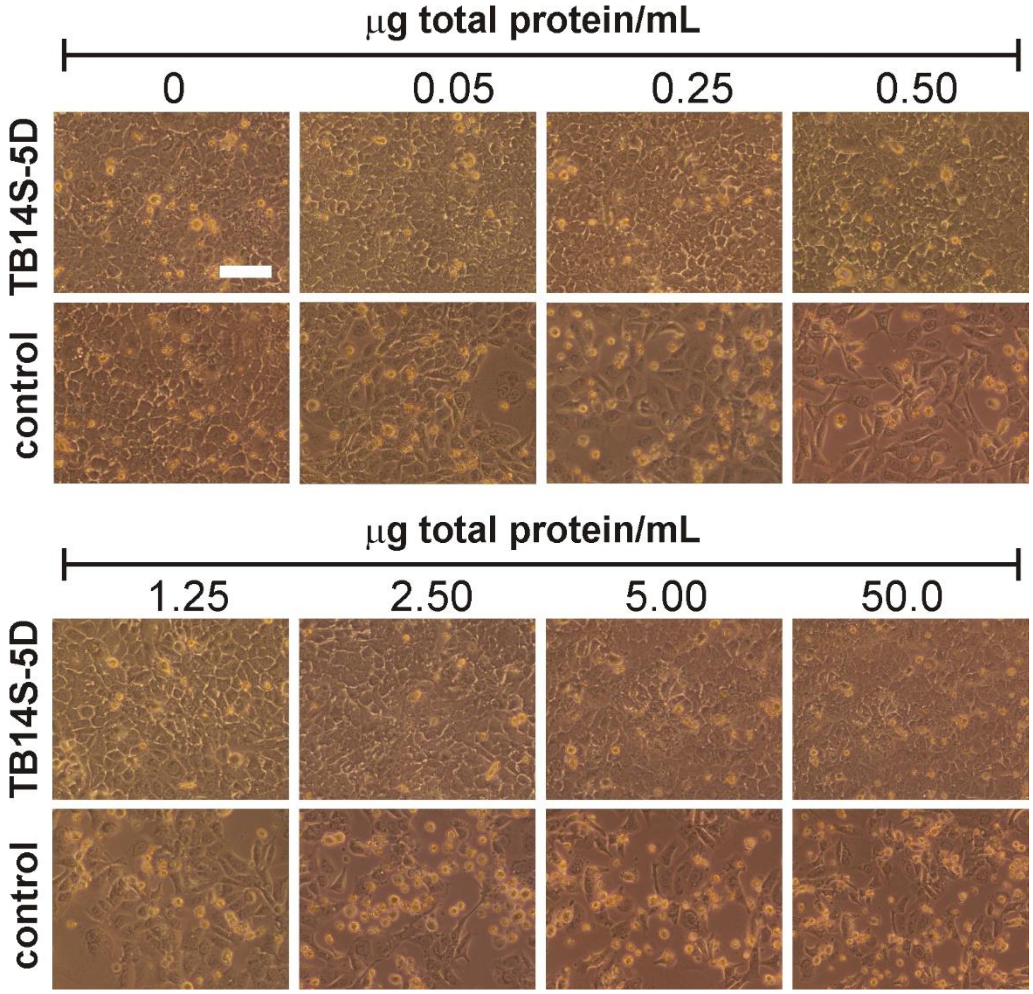


**Supplementary Figure 1.** Small intestine epithelial cells (IEC-6) were exposed to proteins (0 to 50 µg total protein/mL) isolated from endosperm of the transgenic event TB14S-5D and wild type (control). Protein from control plants contains ricin at the concentrations of 0, 1, 5, 10, 25, 50, 100 and 1000 ng ricin/mL. White bar represents 50 µm and magnification is the same in all images.

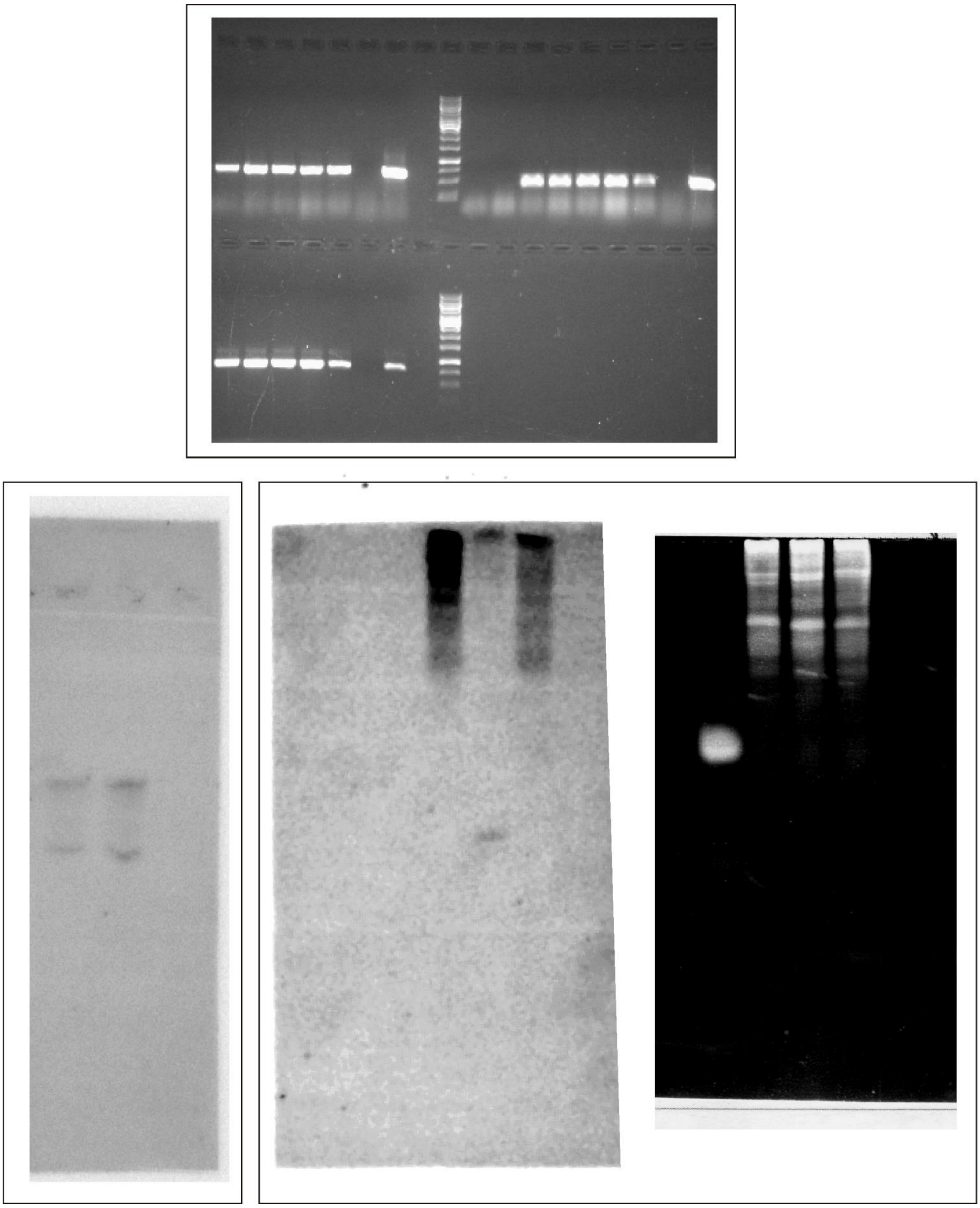

**Supplementary Figure 2.** Full-length gel and blot images used to compose the Figure 1.
